# Mutant *IL7R* collaborates with MYC to induce T-cell acute lymphoblastic leukemia

**DOI:** 10.1038/s41375-022-01590-5

**Published:** 2022-05-17

**Authors:** Mariana L. Oliveira, Alexandra Veloso, Elaine G. Garcia, Sowmya Iyer, Clara Pereira, Vasco M. Barreto, David M. Langenau, João T. Barata

**Affiliations:** 1 Instituto de Medicina Molecular João Lobo Antunes, Faculdade de Medicina, Universidade de Lisboa, Lisbon, Portugal; 2 Molecular Pathology Unit, MGH Research Institute, Charlestown, MA 02129 USA; 3 MGH Cancer Center, Harvard Medical School, Charlestown, MA 02129 USA; 4 Center for Regenerative Medicine, MGH, Boston, MA 02114 USA; 5 Harvard Stem Cell Institute, Cambridge, MA 02139 USA; 6 Smurfit Institute of Genetics, Trinity College Dublin, University of Dublin, Dublin 2, Ireland; 7 DNA Breaks Laboratory, CEDOC - Chronic Diseases Research Center, NOVA Medical School - Faculdade de Ciências Médicas, Universidade NOVA de Lisboa, Lisbon, Portugal

**Keywords:** Acute lymphocytic leukaemia, Oncogenes, Cancer models

## Abstract

T-cell acute lymphoblastic leukemia (T-ALL) is an aggressive pediatric cancer. Amongst the wide array of driver mutations, 10% of T-ALL patients display gain-of-function mutations in the IL-7 receptor α chain (IL-7Rα, encoded by *IL7R*), which occur in different molecular subtypes of this disease. However, it is still unclear whether IL-7R mutational activation is sufficient to transform T-cell precursors. Also, which genes cooperate with *IL7R* to drive leukemogenesis remain poorly defined. Here, we demonstrate that mutant *IL7R* alone is capable of inducing T-ALL with long-latency in stable transgenic zebrafish and transformation is associated with *MYC* transcriptional activation. Additionally, we find that mutant *IL7R* collaborates with Myc to induce early onset T-ALL in transgenic zebrafish, supporting a model where these pathways collaborate to drive leukemogenesis. T-ALLs co-expressing mutant *IL7R* and *Myc* activate STAT5 and AKT pathways, harbor reduced numbers of apoptotic cells and remake tumors in transplanted zebrafish faster than T-ALLs expressing Myc alone. Moreover, limiting-dilution cell transplantation experiments reveal that activated IL-7R signaling increases the overall frequency of leukemia propagating cells. Our work highlights a synergy between mutant *IL7R* and Myc in inducing T-ALL and demonstrates that mutant *IL7R* enriches for leukemia propagating potential.

## Introduction

Acute lymphoblastic leukemia (ALL) is an aggressive and common hematological cancer of childhood. It arises from lymphoid progenitors arrested at different developmental stages, with approximately 15% of pediatric ALL cases being T-cell in origin (T-ALL) and having high risk and poor prognosis [1, 2]. Remarkable improvements in treatment outcome have led to 5-year survival rates reaching 80–90% but require the use of risk-adjusted multi-agent intensive chemotherapy that often leads to both short- and long-term severe complications [3–6]. Furthermore, a significant number of T-ALL cases still relapse and have dismal prognosis. Therefore, it is essential to gain more insight into the molecular mechanisms of T-ALL and its underlying biology to develop novel and more efficient therapeutic strategies that selectively target the leukemic cells while minimizing side effects.

The signaling axis comprised by interleukin-7 (IL-7) and its receptor, composed of IL-7Rα (encoded by *IL7R*) and γc (encoded by *IL2RG*), regulates normal T-cell development and peripheral T-cell homeostasis [7–9]. Inactivation of IL-7 or IL-7R results in severe combined immunodeficiency (SCID) [7, 10–13]. By contrast, constitutive activation of the IL-7/IL-7R axis can induce T-cell leukemogenesis [14], as evidenced by studies showing that transgenic mice overexpressing *Il7* spontaneously develop T-cell lymphomas [15], that IL-7 induces proliferation and survival of human T-ALL cells [16–19] and that IL-7 accelerates disease progression in xenotransplant models of human T-ALL [20]. *IL7R* is also transcriptionally upregulated by Notch [21, 22], one of the most commonly mutated genes in T-ALL [23]. Moreover, somatic *IL7R* gain-of-function oncogenic mutations were identified in approximately 10% of T-ALL patients, including high-risk cases [24–27]. Different studies confirmed that mutant *IL7R* collaborates with *Cdkn2a* deletion, or overexpression of intracellular Notch1 or mutant *NRAS* [28–31], to drive T-ALL. However, the identification of the oncogenic events that cooperate with *IL7R* mutational activation in originating the disease is still limited, and, most important, whether mutant IL-7Rα alone can trigger T-ALL development remains unaddressed. Finally, although there is anecdotal evidence that high IL7R expression correlates with increased leukemia propagating/stem cell activity [22], the impact of *IL7R* activation in regulating the overall frequency of leukemia propagating cells within T-ALL is currently not known.

Here, we show that *IL7R* gain-of-function alone is sufficient to trigger T-cell leukemogenesis in zebrafish, a process that involves increased IL-7R-mediated signaling as well as transcriptional activation of *MYC*. In agreement, mutant IL-7Rα also acts as a collaborating oncogene that synergizes with Myc to drive early-onset T-ALL. Notably, leukemias derived from the combination of *Myc* and mutant *IL7R* exhibit high basal IL-7R-mediated signaling activation and display higher frequency of leukemia propagating cells (LPCs) than T-ALLs derived from *Myc* alone.

## Materials and methods

### Transgenic DNA expression constructs

DNA constructs used to generate transgenic zebrafish have been previously described and included *rag2:mCherry* [32], *rag2:eGFP* [33] and *rag2:Myc* [34]. *IL7R* constructs were created by PCR amplification of the human *IL7R* open reading frame and gateway cloning into the *rag2* promoter destination vector using LR clonase II, according to manufacturer’s protocol (Life Technologies, ThermoFisher 11791020). These *IL7R* constructs harbor two specific mutations found in pediatric diagnostic T-ALL samples, namely *IL7R*^*mut1*^ c.726_727insAACCCATGC (p.L242_L243insNPC) and *IL7R*^*mut2*^ c.731_732insTTGTCCCAC (p.T244_I245insCPT) [24]. PCR primer sequences can be found in Supplementary Table 1.

### Zebrafish T-ALL models

Plasmids were linearized with *NotI* or *XhoI* and column purified. Mosaic transgenic zebrafish were generated as previously described [35, 36]. 40 ng/µL *rag2:mCherry* or *rag2:eGFP* was mixed with 40 ng/µL *rag2:Myc* and 40 ng/µL *rag2:IL7R*^*mut1*^ or *rag2:IL7R*^*mut2*^ and micro-injected into one-cell stage Tu/AB-strain embryos. For experiments in CG1 strain fish, the above mix was diluted 1:1. Stable transgenic *rag2:IL7R*^*mut2*^*-tdTomato* CG1-strain zebrafish were created using the Tol2 transposon system [37]. Animals injected with each DNA construct were randomly selected from a pool of genetically equivalent animals. This initial group allocation as well as data collection and/or analysis were not blinded, since there were no subjective measurements in the experimental analysis. We used the minimum number of animals that allowed to perform standard deviation analysis when required and to achieve statistical significance. Animals were scored for fluorescent-labeled thymocytes at 21- and 28-days post fertilization (dpf) and followed every 7 days for disease onset and progression. Leukemic fish were defined by >50% of their body being infiltrated with fluorescent-labeled T-ALL cells as previously described [38, 39]. Animals that became sick without evidence of leukemia were not considered for downstream analysis. Zebrafish husbandry and help with procedures were provided by MGH Zebrafish Core at MGH, Boston, USA, and by the Zebrafish Unit at iMM-JLA, Lisbon, Portugal. All zebrafish experiments were approved and performed under license 013467/2016, according to the iMM-JLA’s institutional and Portuguese (DGAV) regulations or under animal protocol #2011-N-000127 (Massachusetts General Hospital).

### Histological and immunohistochemical analysis

Fish were sacrificed when moribund and zebrafish leukemias were harvested for further analysis. May-Grünwald Giemsa staining was performed as previously described [34] followed by imaging on the Hamamatsu NanoZoomerSQ, with the help of the Comparative Pathology Unit at iMM-JLA, Lisbon, Portugal. Fixed zebrafish heads were embedded in paraffin, step sectioned, and stained with hematoxylin and eosin (H&E) and TUNEL by the Specialized Histopathology Services at MGH, Boston, USA. Sections were imaged at 400X magnification using an Olympus BX41 compound microscope. The ratio of positively stained cells to total cells was calculated in three separate areas of each head. A square root transformation was applied to each data point to stabilize variance and significance was calculated by Mann–Whitney test.

### Quantitative real-time PCR

RNA was isolated from the cells using the Qiagen RNeasy Mini kit with on-column DNAse treatment, following the manufacturer’s instructions. Total RNA was reverse-transcribed using SuperScript^®^ III First-Strand Synthesis SuperMix (ThermoFisher 11752050) and real-time PCR performed using *Power* SYBR^®^ Green Master Mix in a ViiA™ 7 real-time PCR system (both from Applied Biosystems). qRT-PCR was performed on bulk leukemias or FACS-sorted T-ALL cells (*n* > 3/genotype). PCR primer sequences are available in Supplementary Table 1. Data were normalized to *β-actin* expression and fold-change was calculated using the comparative CT method 2^−ΔΔCT^. Samples were run in triplicate, with error bars representing the SEM of compiled data from all replicates and experimental samples.

### Western blot analysis and antibodies

To evaluate protein expression, cells were lysed in buffer (50 mM Tris-HCl pH 8.0; 150 mM NaCl; 5 mM EDTA; 1% (v/v) NP-40, 1 mM Na_3_VO_4_; 10 mM NaF; 10 mM NaPyrophosphate; supplemented with protease inhibitor cocktail Complete Mini (Roche)), supplemented with 1 mM of AEBSF (Bio-Rad). Total protein was quantified using the Bradford assay (Bio-Rad). Before resolving the protein extracts, samples were resuspended in Laemmli sample buffer (Bio-Rad 1610737) and denatured for 5 min at 95 °C. Equal amounts of protein extracts were resolved by 12% SDS-PAGE, transferred onto nitrocellulose membranes, and immunoblotted with the following primary antibodies: p-STAT5 (Y694) (#9359), p-Akt (S473) (#9271), p-Erk (T202/Y204) (#9101), p-S6 (S235/236) (#2211), STAT5 (#94205), Akt (#9272), Erk (#4695) and S6 (#2217) (all from Cell Signaling Technology) and β-actin (sc-47778 from Santa Cruz Biotechnology). Immunodetection was performed by incubation with horseradish peroxidase-conjugated secondary antibodies (Promega Corporation) and developed by chemiluminescence detection using the Pierce^TM^ ECL Western Blotting Substrate (ThermoFisher). Films exposed to the membranes were developed in a Curix60 (AGFA HealthCare). Amersham^TM^ ECL^TM^ Rainbow^TM^ Marker – Full range (GE Healthcare) was used as molecular weight reference.

### Limiting dilution cell transplantation

T-ALL cells were isolated, transplanted and monitored for tumor growth as previously described [32, 36, 38]. Briefly, tumor-bearing fish were macerated in 5%FBS + 0.9XPBS, cells were strained through a 40 µm nylon mesh (BD Falcon) and isolated by FACS at several dilutions into 96-well plates [36]. Flow cytometry analysis was performed at the MGH Pathology CNY Flow Cytometry Core, MGH, Boston, USA, and at the Flow Cytometry Unit, iMM-JLA, Lisbon, Portugal. Each well contained 50 µL 5%FBS + 0.9XPBS and was supplemented with 3 × 10^4^ whole blood cells isolated from CG1-strain zebrafish. Cells were then centrifuged, resuspended in 5 µL 5%FBS + 0.9XPBS and transplanted into the peritoneal cavity of recipient syngeneic CG1-strain zebrafish. Fish were monitored for T-ALL for up to 120 days. Leukemia-propagating cell frequency and the 95% confidence intervals were calculated using the web-based ELDA (Extreme Limiting Dilution Analysis) statistical software (http://bioinf.wehi.edu.au/software/elda/) [40].

### RNAseq analysis

Leukemia cell pellets were mixed with QIAGEN RLT buffer containing 1% 2-mercaptoethanol, followed by RNA isolation using the RNAeasy Mini kit (QIAGEN). RNA samples were prepped and sequenced on the Illumina HiSeq 2000 platform as previously described [41]. RNA sequence reads were aligned to GRCz10 and the Ensembl version 85. PCR duplicates and ribosomal RNA sequences were removed [41]. Human orthologs were identified using the Beagle web interface and subsequent analysis essentially completed as previously described [41]. RNA sequencing data is available at the Sequence Read Archive (SRA) under accession number PRJNA812715.

### TCR gene rearrangements and clonality analysis

The detection of TCR rearrangements on RNA-Seq raw data was performed with the MiXCR tool [42]. At the time of analysis, the zebrafish *TRB* locus germline sequences were unavailable at the IMGT database. For the alignment, reference sequences were therefore obtained from Meeker et al., 2010 [43], and manually curated for the basic anchor points (FR1 begin, CDR3 begin, and V end – see [42]) and added to the existent latest version of the *Danio rerio* TCR libraries (https://github.com/repseqio/library-imgt/releases) in the json format, with the assistance of the repseqio tool (https://github.com/repseqio/repseqio, doi:10.5281/zenodo.804326). Alignment was performed for the entire library, but only TCR-β productive rearrangements are shown. In order to assess clonal diversity, the relative frequency of each clonotype should be accounted for alongside the clonotypic richness [44]. We have calculated Shannon equitability index, as a measure of clonotype evenness, on a scale from 0 to 1, where 1 represents balanced distribution of clonotypes frequencies, and lower values are indicative of the presence of clonal expansions. MiXCR output tables with clonotype counts were further manipulated and equitability calculated using custom shell and R scripts. TCR rearrangement analysis plots were generated with the R software.

### Statistical analysis

Statistical analyses were performed using GraphPad Prism version 6.01 for Windows (GraphPad Software, CA, USA). Differences between groups were calculated using a two-tailed Student’s *t* test when parametric test assumptions were met. Otherwise, Mann–Whitney tests were performed. Differences in survival curves were analyzed using the Gehan-Breslow-Wilcoxon test. *P* values lower than 0.05 were considered statistically significant.

## Results

### Mutant IL7R alone is capable of inducing T-ALL in zebrafish

**Fig. 1:**
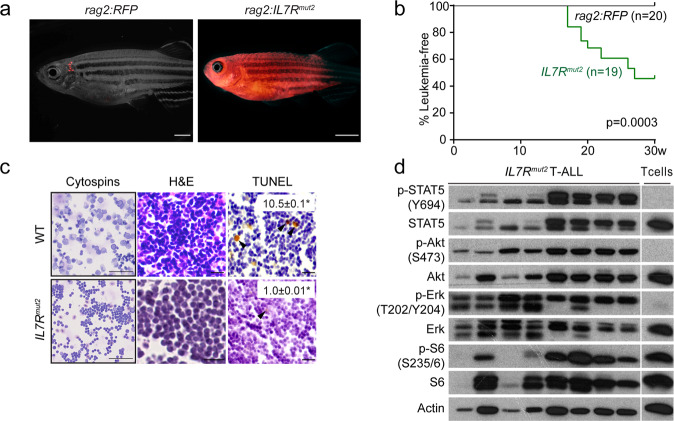
*IL7R* mutational activation alone drives T-ALL in zebrafish. **a** Stable transgenic *rag2:RFP* and *rag2:IL7R*^*mut2*^*-tdTomato* zebrafish were followed for disease onset and progression. Representative images of stable transgenic zebrafish at 17 weeks of life. Panels are merged fluorescent and brightfield images. Scale bar, 2 mm. **b** Kaplan–Meier analysis of disease progression in stable transgenic zebrafish (Gehan-Breslow-Wilcoxon statistic). Number of animals analyzed per genotype is shown in parenthesis. **c** May-Grünwald and Wright-Giemsa stained cytospins of kidney marrow from wild-type fish and bulk leukemias of *rag2:IL7R*^*mut2*^*-tdTomato* fish (left panels); Scale bar, 50 µm. Histological analysis of thymic cells from wild-type fish (*n* = 4) and primary T-ALLs (*n* ≥ 6); Hematoxylin and eosin-stained sections juxtaposed to immunohistochemistry for TUNEL (right panels). Arrowheads denote examples of positively stained cells. Scale bar equals 10 µm. Percent positive cells ± SEM are shown within each image panel. Asterisks denote significant differences as assessed by Student’s *t* test. **d** Immunoblot analysis of phosphorylated protein levels in normal *rag2:RFP* thymocytes and bulk leukemias or FACS-sorted T-ALL cells from stable transgenic animals (*n* = 8).

Somatic *IL7R* gain-of-function oncogenic mutations can be found in approximately 10% of T-ALL patients. Here, we used a stable transgenic approach to explore the capacity of mutant IL-7Rα to trigger T-ALL development. In these experiments all thymic T cells have the potential for transformation over time. Using the Tol2 transposon system [37], we first generated a stable transgenic CG1-strain zebrafish line (*rag2:IL7R*^*mut2*^*-tdTomato*) expressing an *IL7R* mutation (p.T244_I245insCPT) previously identified in a T-ALL patient [24]. This type of *IL7R* gain-of-function mutations, named type 1a [13], are insertions or insertion-deletions in exon 6 leading to the introduction of a *de novo* unpaired cysteine in the juxtamembrane-to-transmembrane region of the receptor that promotes IL-7Rα homodimerization and consequent constitutive signaling [24]. Although none of the mosaic F_0_ founder fish developed tumors (*n* > 100 animals followed for 1.5 years), 9 (47%) out of 19 transgenic F1 offspring developed leukemia with a mean latency of 20 weeks (range of 17 to 27 weeks; Fig. 1A, B). As expected, control stable transgenic *rag2:RFP* animals did not develop disease within their lifespan (Fig. 1B). To characterize mutant *IL7R*-driven leukemias deeper, we next confirmed the lymphoblast morphology of leukemic cells (Fig. 1C) and their T-cell phenotype, as confirmed by qRT-PCR analysis (Fig. S1A). Notably, mutant *IL7R* T-ALLs displayed reduced numbers of apoptotic cells (Figs. 1C and S1B). STAT5, PI3K/Akt/mTOR and MEK/Erk pathways are activated by IL-7R-mediated signaling in healthy lymphocytes and leukemia cells [9, 14, 17, 18, 24, 25, 45–47]. In agreement, we observed hyperactivation of IL-7R-mediated signaling in mutant *IL7R* leukemias, as evidenced by upregulation of phosphorylation levels of STAT5, Akt and S6 (a downstream target of PI3K/Akt/mTOR pathway), and Erk 1 and 2 (Fig. 1D). Interestingly, we observed some heterogeneity in the levels of hyperactivation of each IL7R downstream pathway and found that those samples with milder STAT5 activation tended to display higher relative levels of Erk phosphorylation. Altogether, our data indicate that *IL7R* mutational activation alone can drive T-ALL in zebrafish.

### Mutant IL7R-derived leukemias transcriptionally activate the downstream MYC pathway and are clonal

**Fig. 2:**
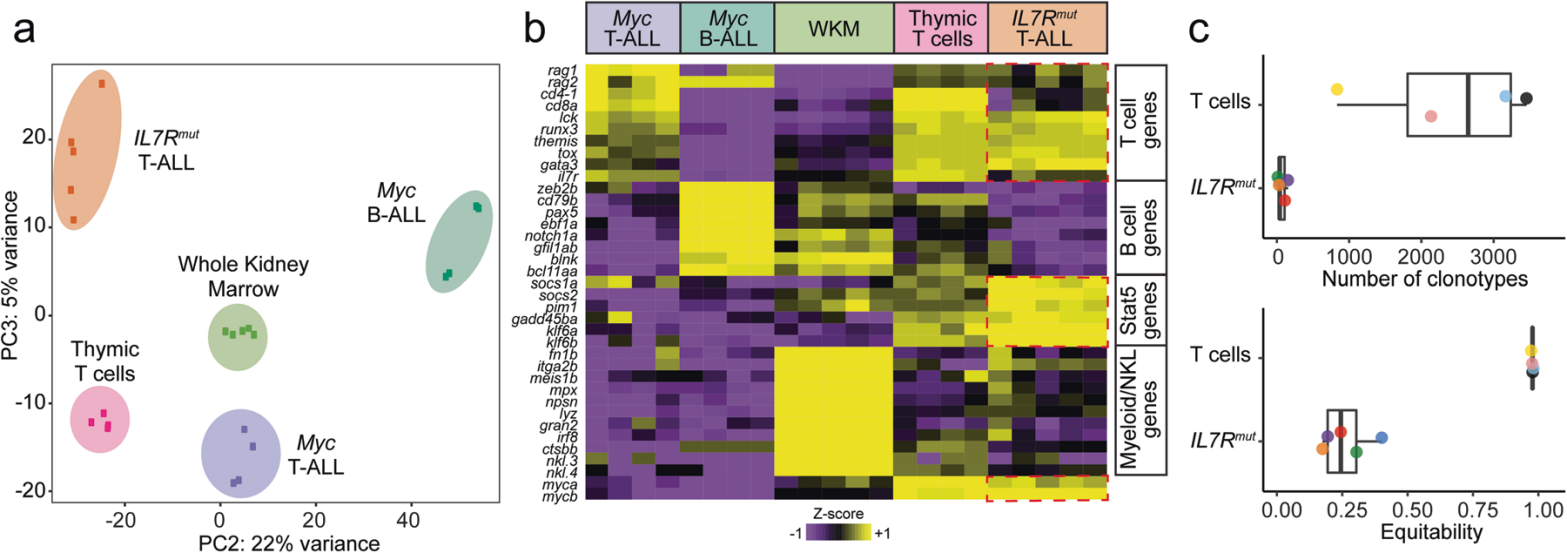
Transcriptomic characterization of mutant *IL7R*-derived leukemias. **a** Principal component analysis (PCA) plot of gene expression profiles from RNA sequencing of different zebrafish leukemias and control samples. **b** Heatmap representation showing expression of well-known T-, B- and Myeloid/NKL-cell associated genes, as well as common STAT5 target genes (adj. *P* < 0.05). WKM, whole kidney marrow. **c** TCR-β gene rearrangements in T-ALLs from stable mutant *IL7R* zebrafish compared with normal thymocytes from control zebrafish. Shown as dotplots and boxplots are the number of clonotypes of the *TRB* locus and the equitability value per sample, both based on productive rearrangements. Higher number of clonotypes indicates higher polyclonality. Higher equitability means the relative frequency of the different clonotypes in a given sample is more balanced, whereas a lower equitability value indicates unbalanced frequencies (i.e. one or a few clones predominate over the others).

We next performed a transcriptomic characterization of primary *IL7R* mutant leukemias. Principal component analysis (PCA) showed that mutant *IL7R*-driven T-ALLs comprise a transcriptionally distinct subgroup that segregates away from *Myc*-induced T- or B-ALLs, as well as from their normal counterparts (Fig. 2A). As expected, RNAseq analysis confirmed qPCR results and showed that mutant *IL7R*-derived leukemias were bona fide T-ALLs (Fig. 2B). Furthermore, analyses of TCR-β gene rearrangements showed that healthy thymocytes displayed a high number of clonotypes with very high equitability values (indicative of normal, highly polyclonal distribution of T cell clones), whereas mutant *IL7R* leukemias displayed few clonotypes with low equitability (Fig. 2C and Supplementary Table 2). This indicates that T-ALLs arising in zebrafish with stable expression of mutant *IL7R* were (oligo)clonal. As expected, mutant *IL7R*-driven leukemias exhibited elevated IL-7R-mediated signaling, indicated by high expression of common STAT5 downstream target genes (Fig. 2B).

Transcriptional alterations in oncogenes and tumor suppressors may potentiate or collaborate with the effects of *IL7R* mutation. We found that *myca* and *mycb* were both upregulated in leukemia cells (Fig. 2B). Together, our results suggest that malignant transformation induced by *IL7R* mutational activation clearly associates with an increase in IL-7R-mediated signaling, evidenced by STAT5 signaling upregulation, as well as with transcriptional activation of *MYC*, an oncogenic driver in human T-ALL [48, 49].

### Mutant IL7R collaborates with Myc to induce early-onset T-ALL in transgenic zebrafish

**Fig. 3:**
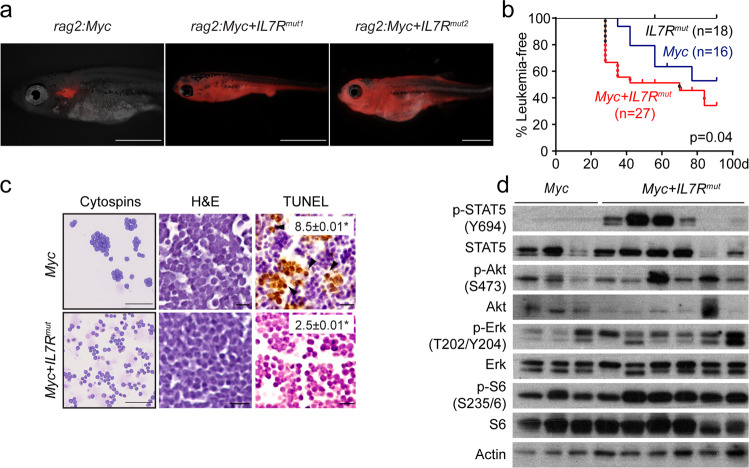
Mutant *IL7R* collaborates with Myc to accelerate T-ALL onset. **a** Tu/AB-strain fish injected at the one-cell stage with either *rag2:Myc* alone or with *rag2:IL7R*^*mut1*^ or *rag2:IL7R*^*mut2*^. Animals were also co-injected with *rag2:mCherry* to visualize leukemia onset and progression. Representative images of transgenic mosaic zebrafish at 28 dpf; Panels are merged fluorescent and brightfield images; Scale bar, 1 mm. **b** Kaplan−Meier analysis (Gehan-Breslow-Wilcoxon test). Number of animals analyzed per genotype is shown in parenthesis. Red dots denote fish that developed leukemia from *rag2:Myc* + *rag2:IL7R*^*mut1*^ injected fish, whereas black dots show leukemias developing in *rag2:Myc* + *rag2:IL7R*^*mut2*^ fish. **c** May-Grünwald and Wright-Giemsa stained cytospins showing lymphoblast morphology (*n* ≥ 2 leukemias/genotype analyzed); Scale bar, 50 µm. Histological analysis of primary T-ALLs (*n* > 3 leukemias/genotype analyzed); Hematoxylin and eosin-stained sections juxtaposed to immunohistochemistry for TUNEL; Arrowheads denote examples of positively stained cells; Scale bar, 10 µm. Percent positive cells ± SEM are shown within each image panel. Asterisks denote significant differences between *Myc* and *Myc* + *IL7Rmut* leukemias as assessed by Student’s *t* test. **d** Immunoblot analysis of phosphorylated protein levels in bulk leukemias or FACS-sorted T-ALL cells (*n* ≥ 3/genotype).

To address the ability of mutant *IL7R* to cooperate with Myc to accelerate the time to leukemia onset, we next used a mosaic transgenic approach to create zebrafish T-ALLs [35, 38, 39, 50, 51]. Specifically, one-cell stage Tu/AB-strain embryos were micro-injected with *rag2:Myc* + *rag2:mCherry* alone or in combination with *rag2:mut2* or another type 1a *IL7R* gain-of-function mutation (*mut1*, p.L242_L243insNPC) found in a different T-ALL patient [24]. Mosaic transgenic fish with mCherry-positive thymus were identified at 21 days post fertilization (dpf) and followed for disease onset and progression. Leukemia was defined as >50% of the fish body being overtaken by fluorescent-labeled cells [38, 39]. These analyses showed that human *IL7R* mutations collaborated with *Myc* (Fig. 3A, B) to accelerate leukemia onset significantly.

Leukemia cells had similar shape and size, with comparable lymphoblast morphology, irrespectively of whether they originated in mosaic *Myc* or *Myc* + *IL7R*^*mut*^ zebrafish (Fig. 3C). As expected, leukemias expressed T-cell specific markers (Lck, CD4, CD8, and TCRα and β), but not B-cell specific genes (e.g. Pax5, CD79a or IgM), indicating they were of T-cell origin (Fig. S1C). Immunohistochemistry analysis showed *Myc* + *IL7R*^*mut*^ expressing leukemias had fewer apoptotic cells than leukemias driven by *Myc* alone, as assessed by TUNEL on section (Fig. 3C, S1D). *Myc* + *IL7R*^*mut*^ leukemias also displayed upregulation of IL-7R-mediated signaling (Fig. 3D). Taken together, our results confirm that mutant *IL7R*, and consequently IL-7R signaling pathway activation, can collaborate with Myc to induce early-onset T-ALL.

### Leukemias arising from the combination of IL7R mutation and Myc overexpression display high basal IL-7R-mediated signaling activation

**Fig. 4:**
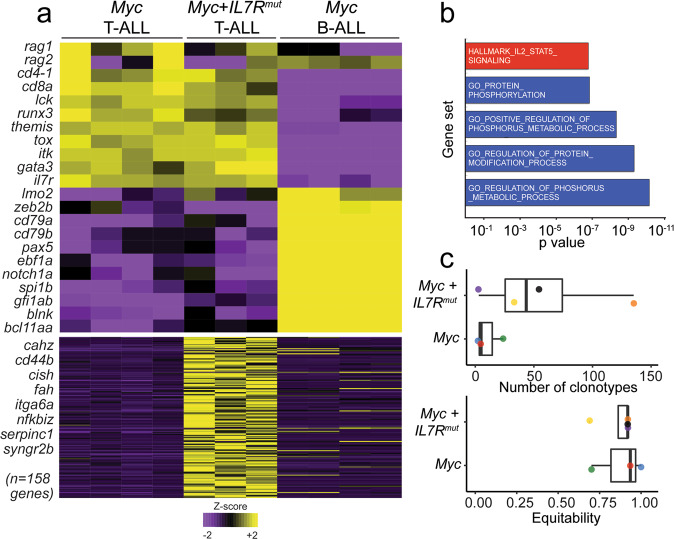
*Myc* + *IL7R*^*mut*^ induced leukemias display IL-7R-mediated signaling upregulation and are polyclonal. **a** Heatmap representation showing expression of well-known T- and B-cell associated genes, as well as common STAT5 target genes (adj. *P* < 0.05). **b** Transcriptome data integration and gene set enrichment analysis show a significant enrichment of the IL-2/STAT5 signaling hallmark gene set in *Myc* + *IL7R*^*mut*^ derived leukemias when compared with *Myc* derived leukemias. **c** TCR-β gene rearrangements in *Myc* + *IL7R*^*mut*^ *vs Myc* derived T-ALLs. Shown as dotplots and boxplots are the number of clonotypes of the TRB locus and the equitability value per sample, both based on productive rearrangements.

We next performed RNAseq on primary T-ALLs to identify potential transcriptional differences between *Myc* and *Myc* + *IL7R*^*mut*^ induced leukemias. As expected from the qRT-PCR results (Fig. S1C), both *Myc* and *Myc* + *IL7R*^*mut*^ expressing leukemias were bona fide T-ALL, as determined by multiple markers of T- and B-cell development (Fig. 4A). Importantly, we observed a unique transcriptional profile in *Myc* + *IL7R*^*mut*^ expressing T-ALLs, including activation of key STAT5 downstream target genes, such as *cish* or *serpinc1* (Fig. 4A and Supplementary Table 3). This reflects high basal IL-7R-mediated signaling activation, as observed also by STAT5 and S6 kinase phosphorylation (Fig. 3D). In agreement, transcriptome data integration together with GO enrichment analysis highlighted the enrichment for the IL-2/STAT5 signaling hallmark gene set in *Myc* + *IL7R*^*mut*^ derived leukemias, as well as for protein phosphorylation (Fig. 4B). Analysis of productive TCR-β gene rearrangements showed that *Myc* + *IL7R*^*mut*^ T-ALLs displayed higher numbers of clonotypes, with similar representation (given by the Shannon equitability index, which accounts for the relative frequency of each clonotype), than leukemias derived from *Myc* alone (Fig. 4C). This implicates that whereas *Myc* T-ALLs tend to be mono or oligoclonal, *Myc* + *IL7R*^*mut*^ T-ALLs tend to be polyclonal (Fig. 4C and Supplementary Table 2). A higher degree of leukemia polyclonality indicates stronger oncogenic potential and transformation of larger pools of initiating cells [31, 45], which is in accordance with the fact that mutant *IL7R* accelerated disease onset in Myc-induced T-ALLs (Fig. 3).

### IL7R pathway activation increases the overall fraction of leukemia propagating cells in Myc-transgenic leukemias

**Fig. 5:**
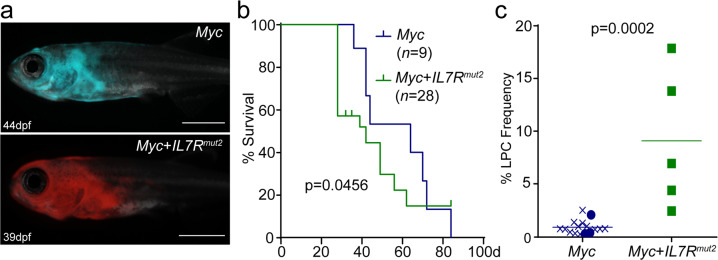
Mutant *IL7R* increases leukemia propagating potential in *Myc*-induced leukemias. **a** CG1-strain fish injected at the one-cell stage with either *rag2:Myc* alone or with *rag2:IL7R*^*mut2*^. Animals were also co-injected with *rag2:mCherry* or *rag2:GFP* to visualize leukemia onset and progression, respectively. Representative images of transgenic mosaic zebrafish at 44 and 39 dpf, respectively; Scale bar, 1 mm. **b** Kaplan–Meier analysis of disease progression (Gehan-Breslow-Wilcoxon test). Number of animals analyzed per genotype is shown in parenthesis. **c** Leukemia propagating cell (LPC) frequency was assessed using limiting dilution cell transplantation analysis and calculated using the ELDA software. Graph showing LPC frequency within *Myc* and *Myc* + *IL7R*^*mut2*^ induced primary T-ALL. Each point represents a distinct primary leukemia generated in this manuscript (filled) and compared with LPC frequency from [38] and [39], denoted by X (Mann–Whitney test). In total, 16 of *Myc*-induced and 5 *Myc* + *IL7R*^*mut2*^ T-ALLs were included in this analysis.

To evaluate whether IL7R modulates leukemia propagating cell (LPC) frequency, we next analyzed the impact of mutant *IL7R* on the self-renewal potential of *Myc*-induced T-ALL cells. We used the transgenic mosaic approach described above to create zebrafish models of T-ALL in CG1 syngeneic fish. Transgenic fish were monitored for leukemia onset and, as in Tu/AB animals, CG1 zebrafish that co-expressed *Myc* and *IL7R*^*mut2*^ developed leukemia faster than those expressing *Myc* alone (Fig. 5A, B). We then performed limiting dilution cell transplantation analyses using primary T-ALLs to determine the frequency of LPCs. As previously demonstrated [38, 39], the mean of LPC frequency in *Myc*-derived T-ALL was 1 in 105 cells (95% CI: 1:77–165), whereas in *Myc* + *IL7R*^*mut*^ leukemias it was significantly higher (*p* = 0.0002, ELDA analysis), with 1 LPC in 11 cells (95% CI: 1:6–97; Fig. 5C and Supplementary Table 4). These results demonstrate that mutant *IL7R* increased the overall pool of LPCs by nearly 10-fold as compared to T-ALLs driven by *Myc* alone.

## Discussion

*IL7R* type 1a gain-of-function mutations introduce a de novo cysteine in the IL-7Rα juxtamembrane-to-extracellular domain, leading to disulfide bridge formation that promotes receptor homodimerization and consequent constitutive downstream signaling [13, 24, 25]. Whether these mutations are sufficient to drive T-ALL remains unclear. Also, the repertoire of oncogenic hits that collaborate to promote T-ALL development in the context of *IL7R* activation is still limited. The mutant allele frequency of *IL7R* mutations in some ALL patients is compatible with *IL7R* activation being an early event in the natural history of the disease [45, 52]. In accordance, our experiments with the stable zebrafish line indicate that mutant *IL7R* alone can be sufficient to trigger T-ALL. Evidently, the relatively low penetrance and long latency of tumor development in this model, together with the clonal nature of the leukemias, indicate that other cooperating hits are required for full transformation and T-ALL establishment. The identification of these hits warrants further investigation. This notwithstanding, our analyses showed that mutant *IL7R* leads to endogenous *Myc* activation, a key player in T-ALL development [34, 53–56], highlighting the importance of the interplay between the two oncogenes in the genesis of this malignancy. In human T-ALL, *MYC* is transcriptionally activated by Notch1 [55, 57], and a majority of T-ALL patients (50–60%) present with *NOTCH1* gain of function mutations [23]. *IL7R* gain-of-function mutations frequently co-occur with *NOTCH1* mutations, and *NOTCH1* mutations tend to be more common in *IL7R* mutant patients (75–90%) than in the general T-ALL population [23, 24, 58]. Moreover, adult T-ALL patients with IL-7R pathway mutations (which associate with Notch pathway mutations) are slow-responders that benefit from post-induction chemotherapy but not from hematopoietic stem cell transplantation [59]. Overall, the cooperative effect we discovered in zebrafish between IL7R and MYC appears to be reflect an interaction that is of biological and clinical relevance in human T-ALL.

Previous studies showed that retroviral expression of *IL7R* mutants in murine T-cell or hematopoietic precursors can collaborate with *Cdkn2a* deletion or with overexpression of mutant *NRAS* (G13D) or intracellular Notch1 to induce T-ALL [28–31]. However, these models require transduction of progenitors in vitro and subsequent transplant into recipient mice, which may limit their physiological relevance. In the present studies, we showed that *IL7R* mutation collaborates with *Myc* in accelerating T-ALL onset and decreasing apoptosis of leukemia cells. Since, contrary to *Myc*, mutant *IL7R* is not sufficient to drive T-ALL in mosaic zebrafish, our results may hint at the possibility that *IL7R* mutation is a late event in T-ALL development which occurs after MYC activation and cooperates with it by preventing apoptosis. This agrees with the fact that *IL7R* mutations in T-ALL patients are often subclonal [58].

Previous studies suggested that IL-7R-mediated signaling may enrich for LPC potential in T-ALL [22, 60]. Here, we provided the first direct evidence, comparing *Myc* versus *Myc* + *IL7R*^mut^ leukemias, that *IL7R* mutational activation increases LPC frequency. Whether this ability is restricted to the collaboration between Myc and IL-7Rα or whether it extends to other oncogenic scenarios should be investigated in the future.

*IL7R* gain-of-function mutations occur not only in T-ALL but also, with lower frequency, in B-ALL patients [25, 61]. Using conditional mutant *IL7R* knock-in mice crossed with CD2-Cre animals to produce progeny in which recombination occurs at the common lymphoid precursor stage, we recently demonstrated that IL-7R activation in lymphoid progenitors leads to the development of B-ALL rather than T-ALL [45]. In these mice, physiological *IL7R* transcriptional regulation is preserved. In contrast, lymphoid-restricted, forced expression of wild type mouse or human *IL7R* drives T-ALL in transgenic mice [14]. Our current studies indicate that ectopic expression of wild type IL-7Rα is not sufficient to promote leukemia development in zebrafish, whereas gain-of-function mutations lead to the development of T-ALL, but not B-ALL. While the exact causes for these differences remain to be determined it seems evident that the ability of *IL7R* to act as an oncogene in B or T lymphoid progenitors will depend not only on *IL7R* mutational status but also on how IL-7Rα expression is regulated. Characterizing these mechanisms and how they impact the sensitivity of particular lymphoid precursors to transformation will be of major importance for the understanding of how *IL7R* partakes in human leukemia development.

T-ALL cases with *IL7R* mutation may benefit from targeted therapeutics against JAK, MEK/Erk pathway, PI3K/Akt/mTOR pathway or BCL2 [14, 19, 62]. Given that IL7R-mediated signaling can confer resistance to glucocorticoids [62, 63] and *IL7R* mutations associate with very poor prognosis upon relapse [27], targeted therapies may be critical to circumvent resistance to conventional therapy and prevent relapse [62, 63]. Our findings are aligned with this possibility. The relevance of the crosstalk between IL7R and MYC activation in the genesis of T-ALL suggests that therapeutic combinations involving bromodomain inhibitors, which can downregulate both *MYC* and *IL7R* [64], may benefit T-ALL cases with *IL7R* mutation and especially those with refractory or relapsed disease [65]. Also, upregulation of Pim1 in T-ALL cells from stable zebrafish is in agreement with previous studies proposing the use of PIM inhibitors in IL-7R-dependent T-ALL, including in relapsed cases [17, 66]. Interestingly, we observe the upregulation of Lck in *IL7R* mutant leukemias, suggesting the possibility that Src-family kinase inhibitors may be of relevance in cases with *IL7R* mutation. In addition, our zebrafish models may be leveraged for the identification of new players in IL-7R-dependent T-ALL and for the fast, economical pre-clinical testing of new targeted therapies against T-ALL.

Overall, our studies demonstrate that *IL7R* mutation is sufficient to trigger T-ALL development in zebrafish, while also cooperating with Myc to accelerate disease onset and increase leukemia propagating cell frequency. They also highlight the potential of zebrafish as a powerful model system to identify cooperating hits in the context of IL-7R-mediated leukemia development and to dissect the underlying molecular mechanisms of how such co-occurring oncogenic hits cooperate in T-ALL development.

## Supplementary information

Supplementary Figure 1

Supplementary Table 1

Supplementary Table 2

Supplementary Table 3

Supplementary Table 4
