# Supplementary material for: Mutant IL7R collaborates with MYC to induce T-cell acute lymphoblastic leukemia
Source: Leukemia. 2022 May 17;36(6):1533–40. doi: 10.1038/s41375-022-01590-5 (PMC9162918; doi:10.1038/s41375-022-01590-5)
Supplement: Supplementary file 1 — Supplementary Figure 1 [file 41375_2022_1590_MOESM1_ESM.pdf]

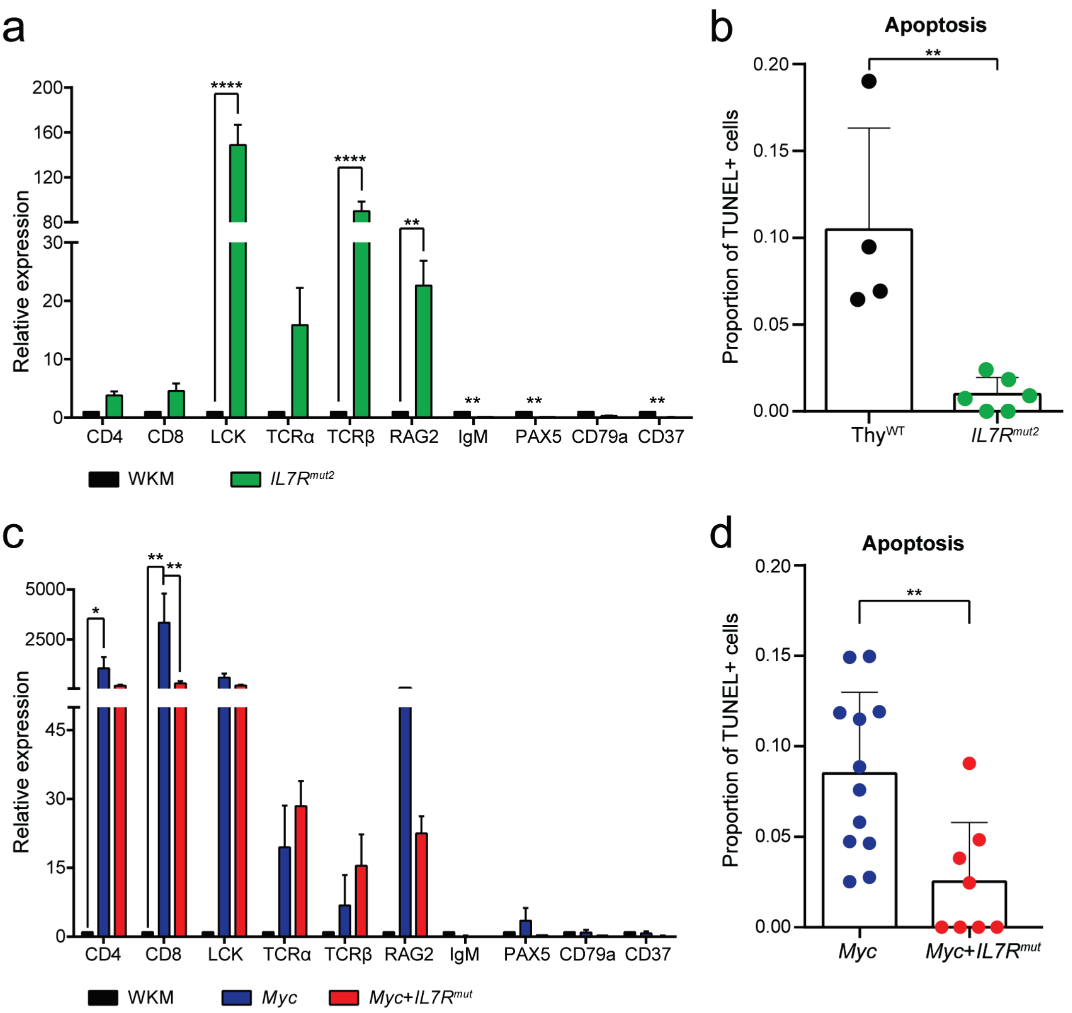

Supplementary Figure S1. Mutant *IL7R* decreases apoptosis of leukemia cells. (a) qRT-PCR gene expression analysis of the T-cell specific genes *lck, CD4, CD8, TCRα*(…), and the B-cell specific genes *IgM, Pax5, CD79a*(…), in tdTomato-positive T-ALL cells from stable transgenic animals (n = 5, run in triplicate); Samples were normalized to *β-actin* expression and gene expression is shown relative to bulk WKM cells isolated from normal CG1-strain fish; **, P < 0.01, ***, P < 0.001 and ****, P < 0.0001, Two-way ANOVA Error bars denote SEM. **(b)** Quantification of IHC data; Each dot represents a thymus or primary leukemia, and mean value is shown; **, P < 0.01; Error bars denote SD. **(c)** qRT-PCR gene expression comparing *Myc* and *Myc+IL7R^{mut}* expressing T-ALLs (n > 3 per genotype, run in triplicate) with bulk whole kidney marrow (WKM) cells isolated from normal Tu/AB-strain fish; *, P < 0.05 and **, P < 0.01, Two-way ANOVA; Error bars denote SEM. **(d)** Quantification of IHC data; Each dot represents a single primary leukemia and mean value is shown; **, P < 0.01, Mann Whitney test; NS, not significant; Error bars denote SEM.
